# RETRACTED: Lin et al. miR-1246 Targets CCNG2 to Enhance Cancer Stemness and Chemoresistance in Oral Carcinomas. *Cancers* 2018, *10*, 272

**DOI:** 10.3390/cancers18152377

**Published:** 2026-07-23

**Authors:** Shih-Shen Lin, Chih-Yu Peng, Yi-Wen Liao, Ming-Yung Chou, Pei-Ling Hsieh, Cheng-Chia Yu

**Affiliations:** 1 School of Dentistry, Chung Shan Medical University, Taichung 40201, Taiwan; sschou@csmu.edu.tw (S.-S.L.); cyp@csmu.edu.tw (C.-Y.P.); rabbity0225@gmail.com (Y.-W.L.); myc@csmu.edu.tw (M.-Y.C.); 2 Department of Dentistry, Chung Shan Medical University Hospital, Taichung 40201, Taiwan; 3 Institute of Oral Sciences, Chung Shan Medical University, Taichung 40201, Taiwan; 4 Department of Anatomy, School of Medicine, China Medical University, Taichung 40402, Taiwan

The journal retracts the article “miR-1246 Targets CCNG2 to Enhance Cancer Stemness and Chemoresistance in Oral Carcinomas” [1] cited above.

Following publication, concerns were brought to the attention of the Editorial Office regarding the integrity of the figures presented in this article [1].

In accordance with the journal’s standard procedures, the Editorial Office and Editorial Board conducted an investigation, which identified evidence of overlapping panels between Figure 2 in [1] and panels in Figure 3 from a previously published article [2], produced by a similar authorship group, representing different experiments. Although the authors cooperated with the investigation and proposed a correction, the Editorial Board concluded that the explanations and the supporting materials provided could not satisfactorily resolve the identified concerns.

As a consequence, the Editorial Board decided to retract this publication in accordance with MDPI’s retraction policy.

This retraction was approved by the Editor-in-Chief of the journal *Cancers*.

The authors have been informed of this retraction and have indicated their disagreement with this decision.
